# Trauma-Related Mortality among Adults in Rural Western Kenya: Characterising Deaths Using Data from a Health and Demographic Surveillance System

**DOI:** 10.1371/journal.pone.0079840

**Published:** 2013-11-07

**Authors:** Frank O. Odhiambo, Caryl M. Beynon, Sheila Ogwang, Mary J. Hamel, Olivia Howland, Anne M. van Eijk, Robyn Norton, Nyaguara Amek, Laurence Slutsker, Kayla F. Laserson, Kevin M. De Cock, Penelope A. Phillips-Howard

**Affiliations:** 1 KEMRI/CDC Research and Public Health Collaboration, Kisumu, Kenya; 2 Centre for Public Health, Liverpool John Moores University, Liverpool, United Kingdom; 3 Department of Parasitic Diseases, Centers for Disease Control and Prevention, Atlanta, Georgia, United States of America; 4 Department of Clinical Sciences, Liverpool School of Tropical Medicine, Liverpool, United Kingdom; 5 The George Institute for Global Health, University of Sydney, Australia; and University of Oxford, Oxford, United Kingdom; 6 India Epidemic Intelligence Service Programme, Centers for Disease Control and Prevention, New Delhi, India; 7 Centers for Disease Control and Prevention, Nairobi, Kenya, Kenya; University of South Florida, United States of America

## Abstract

**Background:**

Information on trauma-related deaths in low and middle income countries is limited but needed to target public health interventions. Data from a health and demographic surveillance system (HDSS) were examined to characterise such deaths in rural western Kenya.

**Methods And Findings:**

Verbal autopsy data were analysed. Of 11,147 adult deaths between 2003 and 2008, 447 (4%) were attributed to trauma; 71% of these were in males. Trauma contributed 17% of all deaths in males 15 to 24 years; on a population basis mortality rates were greatest in persons over 65 years. Intentional causes accounted for a higher proportion of male than female deaths (RR 2.04, 1.37-3.04) and a higher proportion of deaths of those aged 15 to 65 than older people. Main causes in males were assaults (n=79, 25%) and road traffic injuries (n=47, 15%); and falls for females (n=17, 13%). A significantly greater proportion of deaths from poisoning (RR 5.0, 2.7-9.4) and assault (RR 1.8, 1.2-2.6) occurred among regular consumers of alcohol than among non-regular drinkers. In multivariate analysis, males had a 4-fold higher risk of death from trauma than females (Adjusted Relative Risk; ARR 4.0; 95% CI 1.7-9.4); risk of a trauma death rose with age, with the elderly at 7-fold higher risk (ARR 7.3, 1.1-49.2). Absence of care was the strongest predictor of trauma death (ARR 12.2, 9.4-15.8). Trauma-related deaths were higher among regular alcohol drinkers (ARR 1.5, 1.1-1.9) compared with non-regular drinkers.

**Conclusions:**

While trauma accounts for a small proportion of deaths in this rural area with a high prevalence of HIV, TB and malaria, preventive interventions such as improved road safety, home safety strategies for the elderly, and curbing harmful use of alcohol, are available and could help diminish this burden. Improvements in systems to record underlying causes of death from trauma are required.

## Introduction

Globally, injuries account for one quarter of all deaths among people aged 15 to 49 years [1,2]. The public health implications of injuries are well documented in high income countries but have been raised as an important concern also in rural areas of low and middle income countries (LMIC) [3]. Causes of trauma-related death align with gender. Worldwide, injury accounts for approximately half of all mortality among young males aged 10 to 24 years [2]. Available data in LMIC suggest males are at greatest risk of all injury-related deaths, with violence and road traffic injuries (RTI) predominating [3]. Less frequent trauma-related deaths, such as suicide and burns, are also important causes of concern [3,4], and under-documentation of trauma associated with gender-related interpersonal violence has been raised as a public health priority [5].

By 2030, RTI are projected to be the fourth leading cause of death globally [6]. However, the pattern of trauma-related deaths is less well documented in LMIC making it difficult to understand needs or develop and strengthen public health interventions [7-9]. In South Africa, examination of deaths in a rural demographic surveillance site found that injury was responsible for 9% of deaths across all ages, with the predominating causes being homicide, RTI, and suicides [10]. Other types of traumatic injuries in impoverished communities involve living, working and travelling in less safe environments, with few preventative interventions, and minimal access to quality treatment and rehabilitation [11]. In Kenya, as in many other countries of sub-Saharan Africa (SSA), trauma is thought to be an important contributor to premature death in rural communities [12]. However, documentation of trauma deaths outside hospital settings is often limited. Better understanding of the scope and cause of trauma-related deaths in rural areas of SSA is possible through the use of verbal autopsy conducted in health and demographic surveillance systems (HDSS). In western Kenya, the Kenya Medical Research Institute (KEMRI)/US Centers for Disease Control and Prevention (CDC) HDSS provides such an opportunity [13]. This paper reports on deaths attributed to injuries, and poisonings, identified through verbal autopsy, and examines risk factors associated with these deaths.

## Materials and Methods

### Study site and population

The population is described in detail elsewhere [13-15]. Briefly, the study site includes 385 villages spread over a 700km^2^ area along the shores of Lake Victoria, Nyanza Province, western Kenya. The area is rural and includes Asembo, Wagai, and parts of Yala Divisions and from 2008, Karemo Division. The population approximating 220,000 persons, is mostly of the Luo ethnic group, with families living in compounds comprised of a separate house for each wife and her children. The majority of adults are subsistence farmers.

### Health and Demographic Surveillance System (HDSS)

The HDSS site is registered as an International Network for the Demographic Evaluation of Populations and Their Health in Developing Countries INDEPTH partner [13]. Households are geo-spatially located, and the population is registered with each person receiving a unique identification number allowing record linkage. A household census takes place three times a year to capture data on births, deaths, and in and out migration. These census data are used to provide mid-year denominators per five year age group, stratified by gender and area. Identified deaths are followed up by trained field staff to validate and record events around each death using the standard verbal autopsy (VA) methodology. Data are gathered from households on items that provide an indication of wealth status which are then used to calculate a weighted average through multiple correspondence analysis (MCA) [13]. This facilitates the calculation of a composite score ranking of households by quintiles from one (poorest) to five (least poor).

### Verbal autopsy (VA)

VA is conducted at least one month after death using a standardised WHO questionnaire, endorsed by INDEPTH, to gather information on symptoms and care seeking prior to death [16]. Information on risk factors such as alcohol use is gathered in addition to socio-demographic, educational and occupational data. A narrative section of the questionnaire facilitates collection of additional information on events surrounding death. Adult members of the household, preferably the spouse of the deceased, are identified as the most appropriate informants and provide consent for participation. VA forms are reviewed independently by two clinical officers and a single, probable cause of death is assigned, as previously described [17]. In 2007, Sample Vital Registration with Verbal Autopsy (SAVVY) was adopted to strengthen monitoring and measurement [18]. More detailed description on use of VA to examine mortality profiles is published elsewhere [13,15].

### Data preparation and analyses

Adult (15 years and above) data were extracted from the HDSS database for years 2003 to 2008; deaths were grouped into ‘trauma-related’ and ‘non-trauma-related’ using the main diagnosis group code, with underlying cause further examined. Causes of death were categorized under assault, RTI, poisoning, suicide, burns, animal bites/stings, sudden death and ‘other’ trauma-related death with a separate non-specific category for ‘other injuries/poisoning’. Each death was assigned to just one cause of death category. Suicides were recorded within the suicide category irrespective of how the person died (for example a person who drowned themselves would be included in the suicide category only). The category for poisonings refers to accidental only; intentional cases fall under either suicide or assault according to cause. Due to a large number of trauma-related deaths falling into the generic ‘other injury/poisoning’ category, the research team examined the underlying cause of death, enabling re-categorisation of 21 deaths. Three new categories were also created - ‘falls’, ‘drowning’ and ‘other injuries (specified cause)’. Adjustment was made to ensure intentional deaths were included within assault if inflicted on the victim, or suicide if self-inflicted. Deaths were then grouped into intentional (assault and suicide) and unintentional (all other groups) causes. 

Free text was abstracted to provide contextual information on events surrounding trauma-related deaths. Social characteristics, generated through the HDSS, include marital status (ever married, married at death) and school (primary, secondary) attendance. MCA quintiles were aggregated into MCA1-2 (poorest) and MCA3-5 (less poor). Measures for alcohol consumption and for being drunk were dichotomised into regular drinkers (persons reported to have consumed alcohol at least weekly) and non-regular drinkers (occasional drinkers who consumed alcohol less than once a week, including non-drinkers). More detailed breakdown on the frequency of drinking was not possible as four of the six verbal autopsy surveillance years had limited data on patterns of alcohol consumption. 

Analyses were performed using SPSS v17 and EpiInfo Stat Calc. Age at death was categorised and trauma-related mortality rates per 100,000 population were estimated by year, gender and age group using mid-year population estimates from the HDSS census, with 95% confidence intervals calculated. chi-square (χ^2^) tests were used for categorical comparisons. Differences between groups were determined using Pearson's χ^2^ test, with the level of significance set at 0.05 or less. Median age (with interquartile range; IQR) and Mann Whitney U was used to compare male and female ages for each specific type of trauma-related death. Multivariate analysis, using Poisson regression, with adjusted RR, 95% CI, and wald χ^2^, was conducted to identify factors associated with trauma-related deaths; all variables included in univariate analyses were entered into the model. Among duplicate variables presented in univariate analyses, we selected the variable showing strongest association for example, for education - attended primary school; for where died – categorical died at home or elsewhere; for marital status – married at death. Variables thus entered into the model were age, gender, socio-economic status, attended primary school, year of death, married at death, regular alcohol drinker, received care before death, and died at home.

### Ethical considerations

The HDSS protocol and consent procedures, including surveillance and VA was approved by KEMRI (#1801) and CDC Institutional Review Boards (#3308), and is updated annually.

Written informed consent for all compound members to participate in the HDSS activities was provided by compound heads. Any individual could refuse to participate at any time. Named data were securely stored in a MS-SQL database and only authorized data personnel had access rights. Data sets used by scientists for analysis were stripped of names to protect confidentiality.

## Results

**Figure 1:**
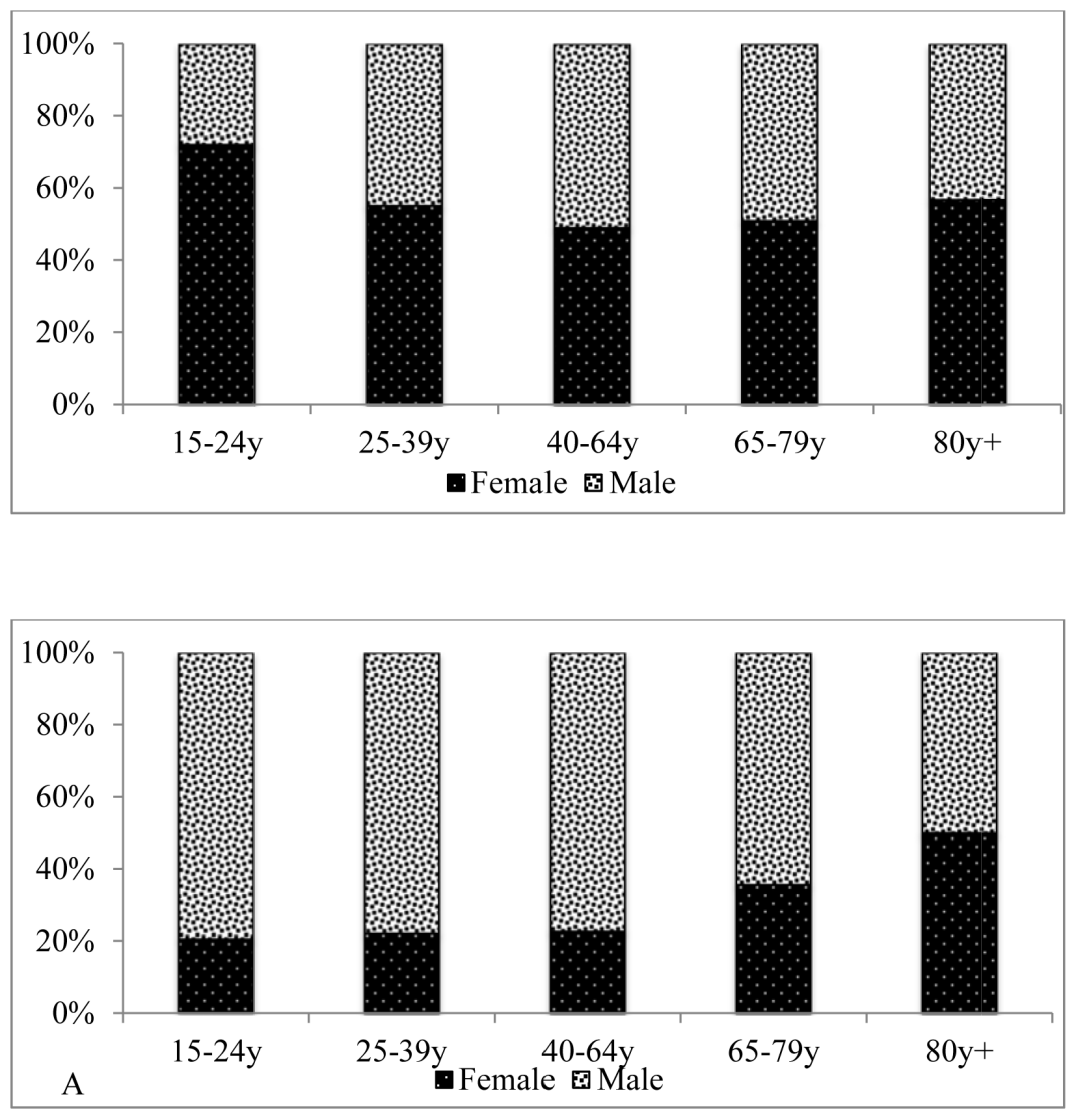
Trauma (panel A) and non-trauma (panel B) deaths among adults aged 15 years and above by age and gender, 2003 to 2008.

**Figure 2:**
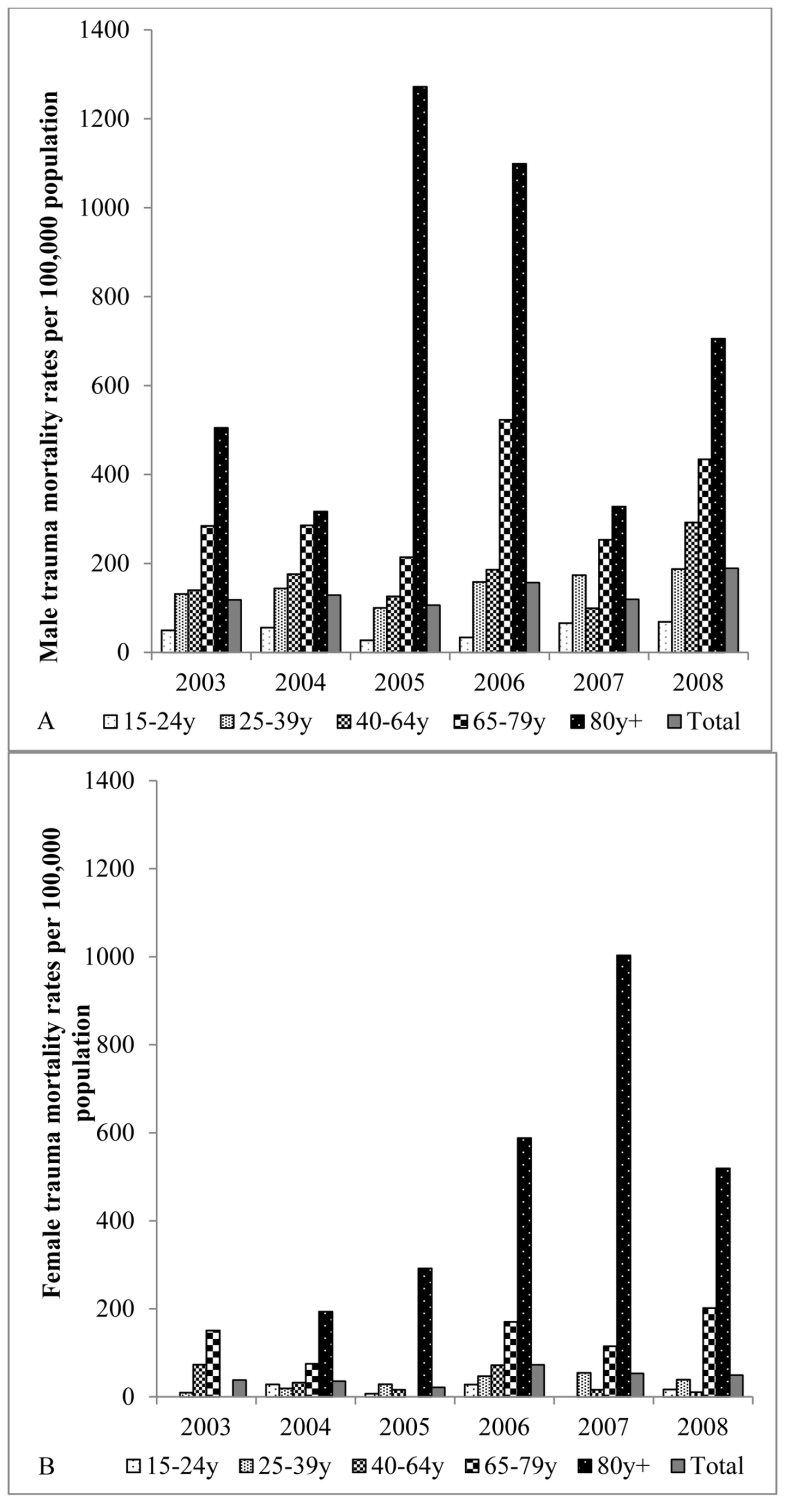
Trauma mortality rates among adults aged 15 years and above by age group and gender, 2003 to 2008. A: Male; B: Female.

Of 11,147 adult deaths recorded between 2003 and 2008, 447 (4% overall, or 2% of female and 6% of male deaths) were attributed to trauma (Tables 1, 2). The proportion of trauma related deaths increased from 2.5% to 5.9% between 2003 and 2008 (test for linear trend 35.5; p < 0.001). Among males, trauma-related deaths were proportionately higher than other causes among adolescents and young adult deaths, with 16% of trauma deaths in persons aged 15-24 years, compared with 5% for all other causes of death (Table 1). In this age group 17% (50/292) of all deaths were trauma related. Among females, trauma-related deaths were proportionately higher than other causes among older aged adult deaths. Except among deaths occurring in the very elderly, trauma deaths were 2 to 4-fold higher among males with, for example, close to four out of every five trauma deaths in the 15 to 24 year old age group being male (Figure 1; Table 3). Overall, mortality rates illustrated a 2 to 5-fold higher burden of trauma-related deaths among males compared with females by age category across the years of study (Figure 2). On a population basis, rates of trauma-related deaths were highest in older persons aged 65 years and above, for both males and females, reflecting the relatively high contribution of trauma amongst the elderly (Figure 2).

**Table 1:** Comparison between characteristics of trauma-related and non-trauma-related deaths among adult males.

Attributes	N	Category	Trauma (%)	Non-trauma (%)	Total (%)	RR (95% CI)	χ^2^ ; p value
Year^1^	5256	2003	39 (12.2)	869 (17.6)	908 (17.3)	Reference	32.7;<0.001
		2004	43 (13.5)	819 (16.6)	862 (16.4)	1.08 (0.87-1.34)	
		2005	36 (11.3)	829 (16.8)	865 (16.5)	0.98 (0.77-1.25)	
		2006	54 (16.9)	781 (15.8)	835 (15.9)	1.23 (1.03-1.47)	
		2007	42 (13.2)	607 (12.3)	649 (12.3)	1.26 (1.01-1.57)	
		2008	105 (32.9)	1032 (20.9)	1137 (21.6)	1.34 (1.21-1.50)	
Age	5256	15 to 24	50 (15.7)	242 (4.9)	292 (5.6)	Reference	67.4;<0.001
		25 to 39	84 (26.3)	1403(28.4)	1487 (28.3)	0.74 (0.64-0.84)	
		40 to 64	95 (29.8)	1796 (36.4)	1891 (36.0)	0.74 (0.66-0.84)	
		65 to 79	61 (19.1)	975 (19.7)	1036 (19.7)	0.69 (0.58-0.81)	
		80+	29 (9.1)	521 (10.6)	550 (10.5)	0.54 (0.40-0.72)	
Area	5256	Gem	159 (49.8)	2726 (55.2)	2885 (54.9)	Reference	8.6; 0.013
		Asembo	124 (38.9)	1858 (37.6)	1982 (37.7)	1.08 (0.94-1.24)	
		Karemo	36 (11.3)	353 (7.2)	389 (7.4)	1.61 (1.18-2.20)	
SES2	4977	MCA1-2	114 (37.3)	1994 (42.7)	2108 (42.4)	Reference	3.5; 0.062
		MCA3-5	192 (62.7)	2677 (57.3)	2869 (57.6)	1.10 (1.00-1.20)	
Ever	4866	No	61 (19.2)	367 (8.1)	428 (8.8)	Reference	45.8;<0.001
married		Yes	257 (80.8)	4181 (91.9)	4438 (91.2)	0.88 (0.83-0.93)	
Married at death	4868	No	113 (35.5)	1567 (34.4)	1680 (34.5)	Reference	0.16; 0.69
		Yes	205 (64.5)	2983 (65.6)	3188 (65.5)	0.98 (0.90-1.07)	
Attend primary3	3336	No	42 (19.5)	703 (22.5)	745 (22.3)	Reference	1.04; 0.31
		Yes	173 (80.5)	2418 (77.5)	2591 (77.7)	1.04 (0.97-1.11)	
Attend secondary	4687	No	241 (80.3)	3644 (83.1)	3885 (82.9)	Reference	1.48; 0.22
		Yes	59 (19.7)	743 (16.9)	802 (17.1)	1.16 (0.92-1.47)	
Any care pre-death	4804	No	147 (52.7)	204 (4.5)	351 (7.3)	Reference	901;<0.001
		Yes	132 (47.3)	4321 (95.5)	4453 (92.7)	0.50 (0.44-0.56)	
Hospital pre-death	4439	No	37 (28.0)	1608 (37.3)	1645 (37.1)	Reference	4.75; 0.03
		Yes	95 (72.0)	2699 (62.7)	2794 (62.9)	1.15 (1.03-1.28)	
Place died	4873	Home	174 (54.9)	3814 (83.7)	3988 (81.8)	Reference	750; <0.001
		HFtravel	10 (3.2)	53 (1.2)	63 (1.3)	3.97 (2.05-7.67)	
		HF	24 (7.6)	261 (5.7)	285 (5.8)	1.89 (1.28-2.80)	
		Hospital	33 (10.4)	394 (8.6)	427 (8.8)	1.70 (1.23-2.36)	
		Other	76 (24.0)	34 (0.7)	110 (2.3)	34.4 (23.4-50.5)	
Regular^4^ alcohol	4727	No	185 (62.5)	3180 (71.8)	3365 (71.2)	Reference	11.6; 0.001
		Yes	111 (37.5)	1251 (28.2)	1362 (28.8)	1.33 (1.14-1.55)	
Regularly^4^ drunk	4236	No	185 (77.1)	3247 (81.3)	3432 (81.0)	Reference	2.56; 0.11
		Yes	55 (22.9)	749 (18.7)	804 (19.0)	1.22 (0.96-1.56)	

^1^ Data collection in Karemo commenced in 2008 ^2^.SES: socio-economic status measured using multiple correspondence analysis in order to calcuate wealth quintiles (1=poorest 5=least poor) ^3^.This question not consistently asked ^4^. Reportedly occurred at least weekly.

**Table 2:** Comparison between characteristics of trauma-related and non-trauma-related deaths among adult females.

Attributes	N	Category	Trauma (%)	Non-trauma (%)	Total (%)	RR (95% CI)	χ^2^ ; p value
Year^1^	5891	2003	16 (12.5)	965 (16.7)	981 (16.7)	Reference	18.9; 0.002
		2004	15 (11.7)	968 (16.8)	983 (16.7)	0.97 (0.67-1.39)	
		2005	9 (7.0)	936 (16.2)	945 (16.0)	0.73 (0.43-1.24)	
		2006	31 (24.2)	929 (16.1)	960 (16.3)	1.35 (1.09-1.66)	
		2007	23 (18.0)	792 (13.7)	815 (13.8)	1.31 (1.00-1.71)	
		2008	34 (26.6)	1173 (20.4)	1207 (20.5)	1.24 (1.02-1.51)	
Age	5891	15 to 24	13 (10.2)	626 (10.9)	639 (10.8)	Reference	25.8;<0.001
		25 to 39	24 (18.8)	1721 (29.9)	1745 (29.6)	0.89 (0.70-1.12)	
		40 to 64	28 (21.9)	1722 (29.9)	1750 (29.7)	0.93 (0.76-1.15)	
		65 to 79	34 (26.6)	1010 (17.5)	1044 (17.7)	1.17 (0.98-1.40)	
		80+	29 (22.7)	684 (11.9)	713 (12.1)	1.32 (1.07-1.63)	
Area	5891	Gem	69 (53.9)	3136 (54.4)	3205 (54.4)	Reference	0.19; 0.91
		Asembo	49 (38.3)	2234 (38.8)	2283 (38.8)	1.00 (0.80-1.24)	
		Karemo	10 (7.8)	393 (6.8)	403 (6.8)	1.14 (0.63-2.04)	
SES2	5513	MCA1-2	66 (55.9)	2850 (52.8)	2916 (52.9)	Reference	0.45;0.50
		MCA3-5	52 (44.1)	2545 (47.2)	2597 (47.1)	0.93 (0.76-1.15)	
Ever	5389	No	13 (10.2)	296 (5.6)	309 (5.7)	Reference	4.74;0.03
married		Yes	115 (89.8)	4965 (94.4)	5080 (94.3)	0.95 (0.90-1.01)	
Married at death	5391	No	93 (72.7)	3382 (64.3)	3475 (64.5)	Reference	3.85; 0.05
		Yes	35 (27.3)	1881 (35.7)	1916 (35.5)	0.77 (0.58-1.02)	
Attend primary3	3982	No	60 (59.4)	1867 (48.1)	1927 (48.4)	Reference	5.03; 0.025
		Yes	41 (40.6)	2014 (51.9)	2055 (51.6)	0.78 (0.62-0.99)	
Attend secondary	5095	No	114 (92.7)	4569 (91.9)	4683 (91.9)	Reference	0.10; 0.75
		Yes	9 (7.3)	403 (8.1)	412 (8.1)	0.90 (0.48-1.71)	
Any care pre-death	5368	No	44 (37.9)	215 (4.1)	351 (4.8)	Reference	283; <0.001
		Yes	72 (62.1)	5037 (95.9)	5109 (95.2)	0.65 (0.56-0.75)	
Hospital pre-death	5080	No	33 (45.8)	1993 (39.8)	2026 (39.9)	Reference	1.08;0.3
		Yes	39 (54.2)	3015 (60.2)	3054 (60.1)	0.90 (0.73-1.11)	
Place died	5398	Home	88 (68.8)	4452 (84.5)	4540 (84.1)	Reference	243; <0.001
		HFtravel	1 (0.8)	75 (1.4)	76 (1.4)	0.68 (0.10-4.82)	
		HF	11 (8.6)	284 (5.4)	295 (5.5)	1.85 (1.05-3.27)	
		Hospital	15 (11.7)	444 (8.4)	459 (8.5)	1.61 (1.00-2.59)	
		Other	13 (10.2)	15 (0.3)	28 (0.5)	38.3 (18.7-78.4)	
Regular^4^ alcohol	5261	No	110 (90.9)	4734 (92.1)	4844 (92.1)	Reference	0.23; 0.63
		Yes	11 (9.1)	406 (7.9)	417 (7.9)	1.15 (0.65-2.04)	
Regularly^4^ drunk	5055	No	108 (94.7)	4710 (95.3)	4818 (95.3)	Reference	0.09; 0.77
		Yes	6 (5.3)	231 (4.7)	237 (4.7)	1.13 (0.51-2.48)	

^1^ Data collection in Karemo commenced in 2008 ^2^.SES: socio-economic status measured using multiple correspondence analysis in order to calcuate wealth quintiles (1=poorest 5=least poor) ^3^.This question not consistently asked ^4^. Reportedly occurred at least weekly.

**Table 3:** Characteristics of trauma-related deaths in adults 15 years and above by gender.

Attributes	N	Category	Female (%)	Male (%)	Total (%)	RR^male^ (95% CI)	χ^2^; p value
Year^1^	447	2003	16 (12.5)	39 (12.2)	55 (12.3)	Reference	7.05; 0.22
		2004	15 (11.7)	43 (13.5)	58 (13.0)	1.08 (0.71-1.65)	
		2005	9 (7.0)	36 (11.3)	45 (10.1)	1.33 (0.75-2.37)	
		2006	31 (24.2)	54 (16.9)	85 (19.0)	0.88 (0.67-1.15)	
		2007	23 (18.0)	42 (13.2)	65 (14.5)	0.88 (0.63-1.23)	
		2008	34 (26.6)	105 (32.9)	139 (31.1)	1.07 (0.87-1.33)	
Age	447	15 to 24	13 (10.2)	50 (15.7)	63 (14.1)	Reference	21.6;<0.001
		25 to 39	24 (18.8)	84 (26.3)	108 (24.2)	0.97 (0.74-1.27)	
		40 to 64	28 (21.9)	95 (29.8)	123 (27.5)	0.96 (0.76-1.22)	
		65 to 79	34 (26.6)	61 (19.1)	95 (21.3)	0.76 (0.60-0.97)	
		80+	29 (22.7)	29 (9.1)	58 (13.0)	0.53 (0.37-0.76)	
Area	447	Gem	69 (53.9)	159 (49.8)	228 (51.0)	Reference	1.37; 0.50
		Asembo	49 (38.3)	124 (38.9)	173 (38.7)	1.06 (0.82-1.36)	
		Karemo	10 (7.8)	36 (11.3)	46 (10.3)	1.46 (0.76-2.79)	
SES2	424	MCA1-2	66 (55.9)	114 (37.3)	180 (42.5)	Reference	12.2; <0.001
		MCA3-5	52 (44.1)	192 (62.7)	244 (57.5)	1.42 (1.14-1.78)	
Ever Married	446	No	13 (10.2)	61 (19.2)	74 (16.6)	Reference	5.37; 0.02
		Yes	115 (89.8)	257 (80.8)	372 (83.4)	0.90 (0.83-0.97)	
Married at death	372	No	80 (69.6)	52 (20.2)	132 (35.5)	Reference	84.5; <0.001
		Yes	35 (30.4)	205 (79.8)	240 (64.5)	2.62 (1.98-3.48)	
Attend primary ^3^	316	No	60 (59.4)	42 (19.5)	102 (32.3)	Reference	50.0; <0.001
		Yes	41 (40.6)	173 (80.5)	214 (67.7)	1.98 (1.55-2.53)	
Attend secondary	423	No	114 (92.7)	241 (80.3)	355 (83.9)	Reference	9.9; 0.002
		Yes	9 (7.3)	59 (19.7)	68 (16.1)	2.69 (1.38-5.25)	
Care pre-death	395	No	44 (37.9)	147 (52.7)	191 (48.4)	Reference	7.2; 0.008
		Yes	72 (62.1)	132 (47.3)	204 (51.6)	0.76 (0.63-0.92)	
Hospital pre-death	204	No	33 (45.8)	37 (28.0)	70 (34.3)	Reference	6.6; 0.01
		Yes	39 (54.2)	95 (72.0)	134 (65.7)	1.33 (1.05-1.69)	
Died	445	No	40 (31.3)	143 (45.1)	183 (41.1)	Reference	7.24;0.007
home		Yes	88 (68.8)	174 (54.9)	262 (58.9)	0.80 (0.69-0.93)	
Regular^4^ alcohol	417	No	110 (90.9)	185 (62.5)	295 (70.7)	Reference	33.5; <0.001
		Yes	11 (9.1)	111 (37.5)	122 (29.3)	4.13 (2.30-7.39)	
Regularly^4^ drunk	354	No	108 (94.7)	185 (77.1)	293 (82.8)	Reference	
		Yes	6 (5.3)	55 (22.9)	61 (17.2)	4.35 (1.93-9.81)	16.9; <0.001

^1^ Data collection in Karemo commenced in 2008 ^2^.SES: socio-economic status measured using multiple correspondence analysis in order to calcuate wealth quintiles (1=poorest 5=least poor) ^3^.This question not consistently asked ^4^. Reportedly occurred at least weekly.

### Causes of trauma-related deaths

Thirty one percent (n=140) of trauma deaths were recorded as arising from intentional causes (assault and suicides), and the remainder were non-intentional. Intentional causes accounted for 37% (n=117) of male deaths and 18% (n=23) of female deaths (RR 2.04, 1.37-3.04, p<0.001). Intentional causes accounted for a similar proportion of deaths among the youngest three age groups (43%, 43% and 39% respectively). Compared to the reference age group of 15<25 years, people aged 65<80 years were less likely to die from intentional causes (RR 0.37, 0.21-0.64, p<0.001), as were people aged 80+ (RR 0.16, 0.06-0.43, p<0.001). When data were split by age group, males were significantly more likely than females to die from intentional causes for ages 15<25 (intentional = 50% of male deaths and 15% of female deaths, RR 3.25, 0.88-11.98, p=0.025) and ages 25<40 (intentional = 49% of male deaths and 21% of female deaths, RR 2.34, 1.04-5.27, p=0.015) but there was no significant difference between males and females in the proportion dying from intentional causes in the older three age groups. 

**Figure 3:**
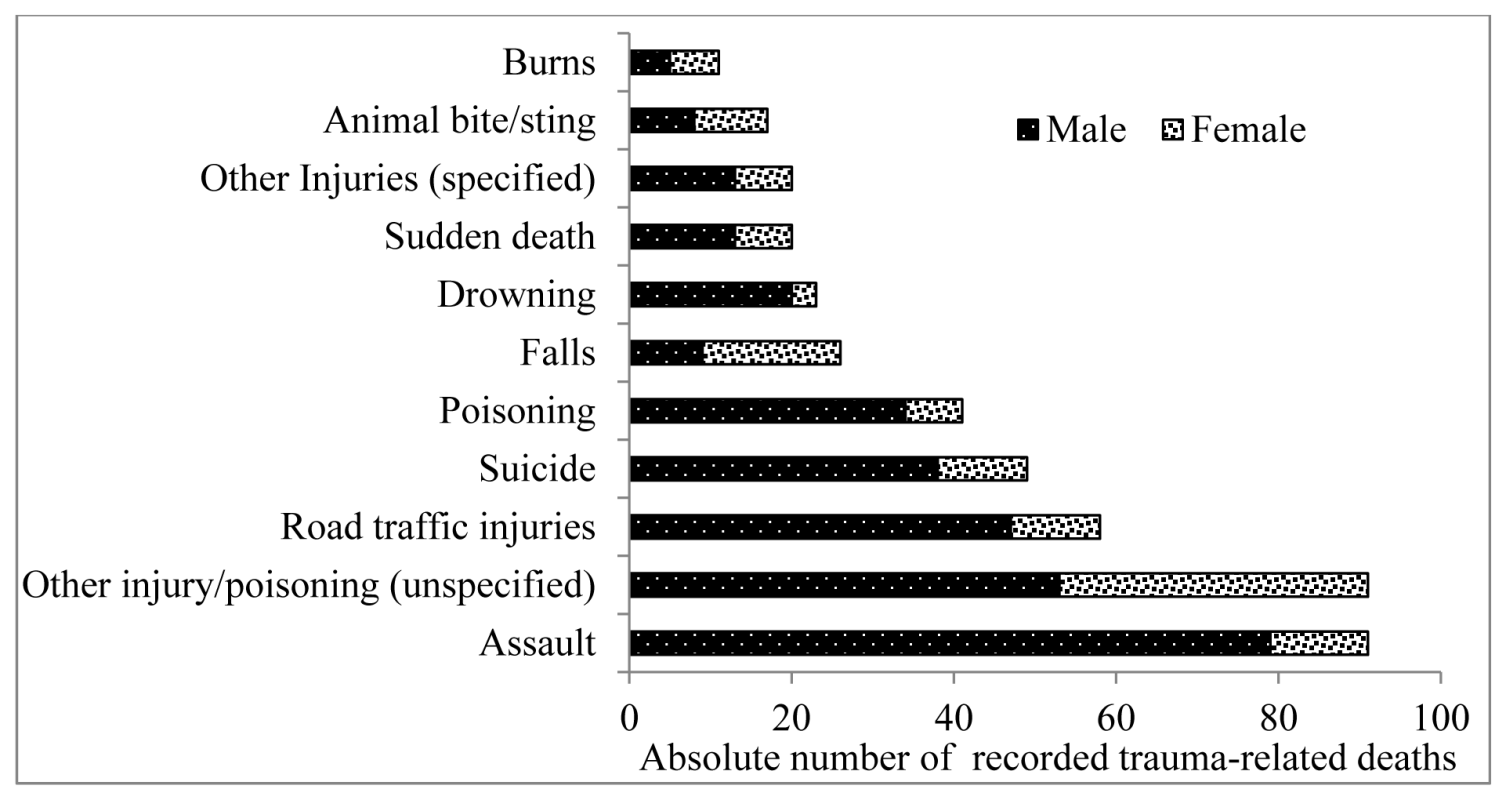
Major causes of trauma deaths among adults aged 15 years and above by gender, 2003 to 2008.

**Figure 4:**
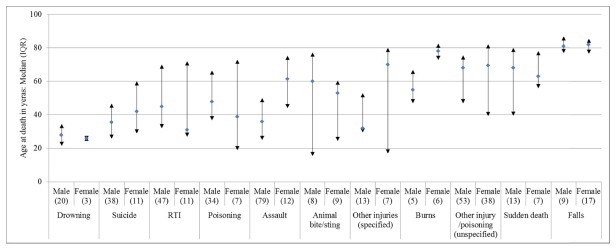
Age at death by cause of trauma death and gender among adults aged 15 years and above, 2003 to 2008.

The leading causes of trauma death among adult females were ‘other injuries/poisoning’ (i.e. unspecified cause of trauma; n=38) and falls (n=17); for males the leading causes were assault (n=79) and RTIs (n=47, Figure 3). The contribution to all trauma-related deaths by cause differed by age and gender (Figure 4). The youngest median age at death for both males and females were among those who drowned, while the oldest median age at death was for males and females who had died from a fall.

### Intentional trauma-related deaths

#### Assault

Among 91 deaths from assault, 79 (87%) were male. Assault contributed 25% of all male trauma-related deaths, except in 2008, when this reached 34%. Deaths clustered in young and middle aged adults, with 54% below 40 years, and 89% below 65 years; deaths occurred among significantly younger males (median age of 36 years (IQR 25-50) compared with females (61.5 years, IQR 44-75; p=0.002). The majority of male assault deaths were from assaults or beatings by individuals or by mobs, with 7 reportedly by a relative, friend, neighbour or the police. Alcohol was documented as a contributory factor in five of the assault deaths; in four of the five deaths both the deceased and the people who attacked them had been drinking. Among those who drank alcohol at least weekly (regular users), 26% trauma-related deaths were caused by assault; for those who drank alcohol less than weekly, 15% of trauma-related deaths were caused by assault (RR 1.8, 1.2-2.6, p=0.006). Six deaths were reported by VA as associated with post-election violence in 2008. Assault-related deaths of females were rarer with 12 reported between 2003 and 2008.

#### Suicide

49 deaths were categorised as definite suicides, of which 38 (78%) were in males. Ninety percent of suicides were among people below 65 years of age, with a median age of 36 years (IQR 26-50.5); there was no difference by gender. Hanging was the main form of suicide, undertaken by 16 males and five females. Fourteen men and four women poisoned themselves. Other means of suicide were drowning (three men and one woman) and burning (one man); with the means of suicide unreported for the remaining five deaths. Forty-five percent of males who died from suicide were regular alcohol consumers, and 3% were reported to get drunk regularly.

### Unintentional trauma-related deaths

#### Road traffic injuries

Of 58 RTI-related deaths, 47 (81%) were in males. Deaths occurred in all age groups, with a third occurring in 25 to 39 year olds; the median age was 43.5 years (IQR 30-70) with no significant difference by gender. Thirty (52%) RTI-related deaths were among pedestrians, 26 (45%) were in drivers or passengers, and the category of the remaining two deaths was unknown. Nineteen percent were regular drinkers, and 9% were reported to get drunk regularly.

#### Accidental Poisonings

Of 41 poisonings, 34 (83%) were in males. Deaths spanned all ages, with 37% below 40 years, and 71% below 65 years, with a median age of 47 years (IQR 34-68) and no difference by gender. Poisoning by alcohol was the main cause, reported in 19 male and two female deaths; it was not recorded whether this was from over-consumption or from tainted local brews. While fewer females died from alcohol poisoning, the circumstances were similar between genders. Sixty-five percent of persons who died from accidental poisoning were regular alcohol consumers, and 41% were reported to get drunk regularly. Among trauma-related deaths who were not regular alcohol consumers, 4% were due to poisonings, but among persons who drank regularly, poisoning accounted for 22% of all trauma-related deaths (RR 5.0; 2.7-9.4, p<0.001).

#### Falls

Of 26 deaths recorded as caused by falls, 17 (65%) were in females, in whom this was the leading specified cause of trauma-related death. Deaths were predominantly in older ages, with 23 (88%) in persons 65 years and above, and 17 (65%) over 80 years; the median age was 81.5 years (IQR 77-87). Falls resulted in broken limbs, back, neck or pelvis, with complications leading to death. Forty-two percent (66% of males, and 26% of females) were regular alcohol consumers, and 29% were reported to get drunk regularly.

#### Drowning

Of 23 deaths due to drowning, 20 (87%) were in males. Deaths were predominantly in young adults, with 83% less than 40 years of age, and a median age of 27 years (IQR 23-33). In three male and all three female deaths the drowning was thought to have occurred as a result of an epileptic convulsion while the person was in or near the water. Two drownings occurred while crossing a river and four were among males who were fishing. Other male deaths occurred in a dam, a well, a stream and the lake. Fourteen percent were regular alcohol consumers, and 5% were reported to get drunk regularly.

#### Burns

Of 11 deaths recorded from burns, six (55%) were in females. Just over half of all burns occurred in persons 65 years or older, with a median age of 72 years (IQR 55-78). Burns were mainly caused by house fires, including persons who caught fire from cooking stoves and lanterns. Fourteen percent were regular alcohol consumers, and 8% were reported to get drunk regularly.

#### Animal bites/stings

17 deaths from bites or stings were distributed equally between males (eight) and females (nine). The median age at death was 53 years (IQR 22-75) with no difference by gender. Of the 14 with a reported cause, eight (57%) were from snake bites, three from dogs, two from bees and one was caused by an unknown insect. All age groups were represented with six (35%) occurring in persons aged 65 and above. Eighteen percent were regular alcohol consumers, and a similar proportion was drunk regularly.

#### Other injuries (specified)

Other specific injuries caused 20 deaths, predominantly in males (n=13, 65%). These occurred in all age groups, with a median of 38.5 years (IQR 28-71); differences in age by gender were not significant. Specific causes of death in this category were varied and included, for example: being struck by lightning or hit by animals (ox, cow, and donkey), electrocution, walls and other structures collapsing onto them, and a gold mining incident.

#### Sudden death

Of 20 persons dying suddenly with no clear cause, two-thirds (65%) were among males. Sudden death was recorded in middle or older age, with 17 (85%) occurring in persons over 40 years, with a median age of 63.5 years (IQR 50-78). Information around the deaths was sparse; in six cases the informants could not say how the death had arisen as they were absent when the death occurred. Other records described sudden death as related to old-age.

#### Other injury/poisoning (unspecified)

This category, consisting of 91 deaths, 53 (58%) in males, was distributed across the age groups. The median age at death was 69 years (IQR 43-78), with no difference by gender. In the majority of these cases, information available did not allow a specific cause to be attributed. Over half (n=52, 57%) were among people aged over 65.

### Other characteristics of trauma-related deaths

For both males and females, people dying from trauma were significantly less likely than those dying from other causes to be recorded by the VA as receiving medical care after the trauma and before death (both p<0.001, Table 1). Among trauma-related deaths more females had sought any form of care from both formal and informal health sectors including traditional sources prior to death compared with males (62% vs 47%; p=0.008). However, a significantly lower proportion of females received formal medical care from a hospital (54% compared with 72% among male deaths; *p*=0.01), while a significantly higher proportion of females (69%) compared with males (55%), died at home p=0.007, Table 3).

Males dying from trauma were significantly more likely to be regular alcohol consumers (38%) compared to those dying from other non-trauma-related causes (28%; p<0.001, Table 1). Of trauma-related deaths, males were significantly more likely than females to consume alcohol regularly (RR 4.1, 2.3-7.4) and to get drunk regularly (RR 4.4, 1.9-9.8, Table 3).

### Multivariate analysis

Each key variable shown to be significant in univariate analysis contributed to the multivariate analysis (Table 4). Gender, age, alcohol consumption, receipt of any care prior to death, and place of death (home or health facility) remained significant within the model. Socio-economic status, education (primary school attendance), year of death, and marital status were not significant. The risk of death from trauma rose with age, with persons aged over 80 and persons 65-80 years, 7- and 4-fold more likely to be ascribed trauma as their cause of death, compared with young adults (Table 4). Males had a 4-fold higher risk of a trauma-related death (ARR 4.0, 1.7-9.4) than females. Regular alcohol consumption remained significantly associated with trauma-related deaths (Adjusted RR 1.5, 1.3-1.9). The strongest predictor of a trauma death was absence of any care prior to death (ARR 12.2, 9.4-15.8). Despite this, trauma cases were 3-fold more likely to die outside the home reflecting the frequency of RTI and other fatalities.

**Table 4:** Factors predicting trauma-related deaths, multivariate analysis (n=6076 deaths).

Attribute	Category	Exp (B)	95% Confidence interval	p value
Year^1^	2003	Reference		
	2004	0.93	0.59-1.49	0.78
	2005	0.71	0.42-1.21	0.21
	2006	1.35	0.88-2.10	0.17
	2007	1.15	0.74-1.80	0.53
	2008	1.30	0.83-2.02	0.25
Sex	Female	Reference		
	Male	3.99	1.71-9.39	0.001
Age (years)	15 to 24	Reference		
	25 to 39	1.41	0.55-3.67	0.48
	40 to 64	1.65	0.52-5.29	0.40
	65 to 79	4.23	0.84-21.24	0.08
	80+	7.33	1.09-49.19	0.04
SES^2^	MCA1-2	Reference		
	MCA3-5	1.25	0.96-1.64	0.10
Attend Primary	No	0.97	0.65-1.43	0.86
School	Yes	Reference		
Married	No	0.96	0.72-1.28	0.78
at death	Yes	Reference		
Any care	No	12.21	9.43-15.80	<0.001
	Yes	Reference		
Died at home	No	3.00	2.25-3.92	<0.001
	Yes	Reference		
Regular^3^ use of alcohol	No	Reference		
	Yes	1.46	1.13-1.89	0.004

^1^ Karemo excluded as data collection commenced in 2008 ^2^.SES: socio-economic status measured using multiple correspondence analysis in order to calcuate wealth quintiles (1=poorest 5=least poor). Interaction terms sexF*age groups 80y+ (expB 0.23); 65<80y (expB 0.37); 40<65y (expB 0.45); 25<40y (expB 0.53); 15<25y (reference) ^3^ Reportedly occurred at least weekly.

## Discussion

The burden of trauma is growing globally and there has been a call for improving surveillance methodologies to capture information for public health interventions [6]. In this paper we present unique data identifying deaths associated with trauma in a rural community of Eastern Africa. While trauma contributes a relatively small percentage of deaths in rural areas of Eastern and Southern Africa (here 2% of female deaths and 6% of male deaths), where a high burden of HIV, TB and malaria dominate, case histories show considerable suffering and lack of access to care, in particular hospital-based care for women.

Global estimates suggest injuries account for half of all male mortality in the ages 10 to 24 [2,3]. Here, the contribution of trauma to all deaths in young adult males aged 15 to 24 years is lower (17% of all male deaths in this age group), but still considerable. Separate analysis found trauma equalled HIV as the main contributor to deaths among young males in this area [15], and confirms the need to document such health threats in adolescents and young adults in SSA [19]. We noted physical injuries leading to death among elderly members of the community; on a population basis, trauma-related mortality rates were highest among persons 65 years and older of both genders. While this belies the larger number of trauma deaths numerically occurring in younger age groups, it indicates a need for interventions to reduce injury risk in this age group, for example, home assessment and reducing safety risks.

Deaths from intentional causes accounted for one third of all deaths; of these there were 91 deaths from assault and 49 from suicide. Deaths from intentional causes accounted for a greater proportion of male than female deaths and a greater proportion of deaths of people in the youngest three age groups than the oldest two. Indeed assault was the main cause of death from trauma among males, comprising 25% of all male trauma deaths and 36% of deaths among young males aged 15 to 24. Interpersonal violence contributed 5.2% of all deaths among males aged 15-49 years globally in 2010 [2], with 12% of deaths among young males (ages 10 to 24 years) estimated to be due to violence [3]. In South Africa, interpersonal violence was the second leading cause of healthy years of life lost and Norman and colleagues recommended that violence be considered a priority health problem as well as a human rights and social issue [20]. In 2008, the number of male deaths from assault occurring in the HDSS accounted for 34% of male deaths, likely related to post election violence, raising the need for protective interventions during election periods when civil unrest might be predicted. 

Global Burden of Disease data highlight injuries as an important cause of death in females, as well as males, worldwide [2-4]. Among females, injury from assault is mainly associated with intimate partner violence (IPV), with 1.6% of deaths among females 15-45 years due to this cause worldwide in 2010 [2]. However, prevalence studies show endemic levels of IPV in many countries [22]. In the nationally representative Kenyan Demographic Health Survey, 25% of women reported having been violently abused in the 12 months prior to survey [23]. Highest rates (of 36%) were reported in Nyanza Province, where the HDSS is located. Reasons women gave for “deserved beatings” included burning food, arguing, going out without telling, child ‘neglect’, and refusing sex [23]. However, despite the reported pervasive violence, only 1% of adult female deaths in the HDSS were classified as assault. Under-ascertainment of deaths from IPV is suspected since documentation through VA depends upon the spouse or partner, as next of kin, to report on the events surrounding death. Further consideration should be given to how IPV can better be captured using the VA tool.

The 49 deaths in the current study from suicide accounted for 11% of all injury deaths. By comparison, in South Africa, 9% of all injury deaths were due to suicide [20]. Globally, suicide (self-harm) was identified as a leading cause of death for both males and females in 2010, contributing 5.7% and 4.8% of all deaths in this age group, respectively [2]. These data underscore the need to strengthen health care services in LMIC for mental health disorders, including depression, which is the leading global cause of years lived with disability [21].

Between 1990 and 2010, one million more deaths from injuries worldwide has been calculated (a 24% increase), and accounting for close to one in ten (9.6%) of all deaths [2]. This was largely due to a rise in unintentional injuries, for example a 46% rise in deaths from road traffic injuries, and an increase in deaths from falls [2]. In our study area, RTIs accounted for 58 (13.0%) of trauma-related deaths; 81.0% were among males, a third occurred in persons aged 25 to 39, and half of the deaths were among pedestrians, who tend to walk on roads rather than footpaths. The contribution of RTIs to mortality in Kenya is significant in terms of human suffering and financial cost, and the number of fatalities is increasing [24,25]. Pedestrians and passengers are most affected [25,26], and in rural areas, passengers in buses and public service commuter vans (*matatus*) account for the majority of fatalities [24,25]. Road safety is determined by both individual behaviour and structural issues [25,26], and both should be considered by the National Road Safety Council of Kenya in terms of formulating road safety policy. Such policies could include the development of footpaths to protect pedestrians, education about where to walk and the need for reflective clothing if walking at night, street lighting, proper road maintenance, speed enforcement and regulations that ensure that drivers are capable of driving safely and vehicles are road worthy [26]. That trauma deaths significantly occur outside of care, and at home, also suggest road accident victims do not reach services in time to receive life-saving treatment, and support is needed to develop ambulance services to reach persons critically injured.

We identified a significantly higher proportion of male trauma-related deaths among regular alcohol drinkers compared with non-regular drinkers. Alcohol consumption is a well-established risk factor for injury [27]. Alcohol consumption in this population is characterised by persons drinking until they get drunk [28], placing them at risk of injury. Nearly half of all male assault deaths and a third of all female assault deaths recorded were regular consumers of alcohol. Four out of every five male deaths from poisoning were also reported to be regular drinkers. Poisoning may arise from excessive ethanol consumption or from tainted brews. Separate systematic chemical analyses of locally used traditional homebrews in Kenya found that an average *changaa* (a traditional spirit) drink equalled 3.5 standard drinks in the UK (2 in the USA), and *busaa* (a traditional beer) equalled 2.3 (1.3 in the USA) [29]. The ethanol content of *changaa* varied from less than 20% to just over 50% [29]. Toxicity from contaminants in the brewing process, by error or to enhance alcohol content, cause additional and potentially large scale morbidity and mortality [30]. The Kenyan Alcoholic Drinks Control Act, introduced in 2010 to control illicit brewing [31], has yet to be examined to determine its full impact on trauma-related morbidity and mortality.

Our multivariate analysis illustrated the risk of death among the elderly, persons who died without receiving any form of care, and who died in the home. Our data also show how unintentional injuries from falls disproportionately affected the elderly and women. Each year, about a third of people aged 65 years and over experience a fall, although this varies by country [32]. Unintentional falls among the elderly result from environmental hazards and an increased susceptibility due to sight loss, memory impairment, poorer posture control and muscle strength and tone [33]. Lower educational level and lack of resources place the elderly at risk of injury, and limit their ability to seek care following injury. Regular alcohol consumption was reportedly higher among these female deaths; a survey among this population noted the frequency of alcohol consumption increased with age among females, potentially placing the elderly at risk of accidental injuries [28]. This community, as in many SSA countries, has been disrupted by deaths from HIV/AIDs in prime aged adults, frequently leaving elderly female widows in poverty and unsupported [34]. The VA documented deaths following broken limbs, back, neck and pelvis as a consequence to falls, and found few of the injured received care from hospital after their fall and prior to death. Coupled with the deaths from house fires, especially among older women, these results suggest the need for home assessments to develop safety strategies through Kenya’s community health worker programme.

### Study limitations

This study has a number of important limitations. First, the population has been part of a health and demographic surveillance system for over a decade and thus, has been exposed to numerous trials testing interventions. These have all been for communicable disease control, however; and impact on trauma-related deaths is likely to be relatively small. Second, the number of deaths attributed to trauma constitutes only a small proportion of all deaths in rural communities affected by HIV, TB, and malaria, minimising the scope of analysis possible. Third, while the sensitivity and specificity of both physician and computer algorithm based diagnoses are higher for injuries than any other cause of death [35], we acknowledge that trauma-related deaths may be misclassified. Questioning of the closest relative on events surrounding death has severe limitations, particularly for ascribing cause of death for trauma. Literature illustrates that a high proportion of deaths from intentional injury are perpetrated by an assailant known to the victim; yet in our data a very low proportion of female deaths were ascribed to IPV, despite a known high prevalence of domestic violence in this population. False reporting of cause of death requires additional research in order to adjust for this important public health concern. Moreover, one in five deaths lacked the details necessary to ascribe a specific trauma-related cause; because of the relatively small number of deaths attributed to trauma, lack of information among 20% of deaths severely hampered more sophisticated analyses. This may have also occurred among unintentional trauma deaths for more benign reasons, such as family migration, observer recall bias, and no living relative. This is particularly relevant for deaths among the elderly who may have no living relative close by, or whose spouse is infirm and unable to adequately respond. We suggest follow-up of deaths occur within a narrower time window to minimise migration loss and reduce recall bias. Approaches to generating information on vulnerable deaths, however, clearly require further exploration. Lack of data on cause of death extends also to minimal information being captured on a range of data characterising the individual. Alcohol consumption, socio-economic status, age and gender, and marital status have been available, but other information is only available through informal reporting, such as fighting or social pressures occurring before the fatal event. The VA questionnaires have hitherto included open text fields, allowing the recording of consequences such as “fell and broke neck”, “clothes caught fire from cigarette”, or “fall resulted in complications leading to death”. These have been useful in attribution, and we are reluctant to endorse attempts to remove textual information from VA questionnaires. Finally, underlying pathology in many of such deaths may not have been recognized by the next of kin, resulting in only the physical consequences being recorded; for example, falls among the elderly may be caused by hypotension, ischaemic heart disease, or stroke. While VA classifies ‘sudden death’ as a trauma death; cerebral vascular deaths or cardio-vascular disease resulting in sudden death may again be misclassified as trauma. Due to these limitations, we have chosen to limit the scope of our analysis to descriptive characterisations, and believe these data contribute towards an understanding of the causes of trauma, current pitfalls in knowledge, and inferences for research and preventive interventions to minimise such deaths. 

## Conclusions

Publications documenting deaths from trauma, especially in LMIC, are relatively scarce. However, an understanding of causes of largely preventable trauma deaths is needed to guide the design of public health interventions and policies [36]. We conclude that, while a small portion of deaths in rural areas with high HIV, TB and malaria prevalence, are ascribed to trauma they represent preventable mortality, and interventions to reduce these deaths are urgently needed. Trauma among young males is well documented, but surprisingly high in this rural population, matching the mortality from HIV [15]. Here we also show the vulnerability of older groups, especially older women, from trauma deaths such as falls. Data suggest the elderly subsequently die at home, without any resort to care. Policies and guidelines for targeting interventions to prevent trauma in different population groups are needed, as are improvements in systems and tools to record underlying causes of death associated with trauma. Methods are required to improve the quality of data, including the underlying causes of deaths; while targeted research could provide new insights, more logistical changes such as earlier follow-up, and a broader scoping of informants could contribute to strengthening systems. Potential under-reporting of deaths from intimate partner violence among women is a particular concern. 
